# Topography and relationship-specific social touching in individuals displaying body image disturbances

**DOI:** 10.1038/s41598-023-39484-w

**Published:** 2023-08-14

**Authors:** Ashleigh Bellard, Jyothisa Mathew, Wenhan Sun, Linda Denkow, Ali Najm, Despina Michael-Grigoriou, Paula Trotter, Francis McGlone, Merle Fairhurst, Valentina Cazzato

**Affiliations:** 1 Faculty of Health, Research Centre for Brain and Behaviour, School of Psychology, Liverpool John Moores University, Liverpool, United Kingdom; 2 Department of Psychology, Bundeswehr Universität, Munich, Germany; 3 Faculty of Philosophy and Philosophy of Science, Munich Center for Neuroscience, Ludwig Maximilian University, Munich, Germany; 4 GET Lab, Department of Multimedia and Graphic Arts, Cyprus University of Technology, Limassol, Cyprus; 5 Institute of Psychology, Health & Society, University of Liverpool, Liverpool, United Kingdom; 6 Faculty of Electrical and Computer Engineering, Centre for Tactile Internet With Human-in-the-Loop (CeTI), Technische Universität Dresden, Dresden, Germany

**Keywords:** Human behaviour, Sensory processing

## Abstract

Interpersonal touch is intimately related to the emotional bond between the touch giver and the touch receiver. Which bodily regions we touch in those individuals in our social network is relationship specific. Perception of interpersonal touch is altered in psychiatric disorders characterised by body image disturbances (BIDs). Here, we examined whether the ‘imagined’ experience of social touch in individuals with BIDs is body topography- and relationship-specific. By using an interactive media mobile App, the *Virtual Touch Toolkit*, high versus low levels of BIDs participants completed heatmaps of full-body virtual avatars, to indicate the body regions they find soothing/unpleasant to be touched by a loved one versus an acquaintance. Self-reports of interoceptive awareness and dysmorphic concerns were also measured. Overall, imagined touch was rated as the most soothing when received from a loved one, and also when this was delivered to ‘social’ body regions. The importance of the social relationship for the imagined tactile interactions was particularly evident for the high levels of BIDs group, with greater problems with interoceptive awareness predicting higher soothing touch ratings when this was received by a loved one. Despite the evidence that imagined bodily contacts between meaningful people is the most pleasant for socially acceptable bodily regions, our findings may suggest a greater sensitivity to relation-specific bodily patterns of social touch particularly in the high level of BIDs group. Heightened interoceptive awareness may also play a key role in this experience of bodily affective contacts. Future research for body-oriented therapy for BIDs is encouraged to systematically probe the efficacy of imagined social touch interaction protocols which use more plausible, ecological, scenarios where touch is delivered by loved ones and to socially acceptable bodily regions.

## Introduction

Social touch is a powerful nonverbal behaviour exchanged between socially significant persons to communicate emotions and affection^1–5^. Pleasantness and comfort derived by social touch is believed to benefit individuals in many ways. Friendly physical contact is experienced as pleasurable and is hypothesised to be due, in part, to the stimulation of a population of unmyelinated, low-threshold mechanosensory C-tactile afferents (CTs) that respond optimally to slow, gentle touch to the hairy part of the skin, and processed in the Insula Cortex, a region involved in the affective dimension of touch^6–8^. Moreover, touch induces positive psychophysiological changes to the touch receiver, such as a reduction in blood pressure, reduced anxiety, and a decrease in heart rate^9–11^, all of which are important for an individual's mental and physical wellbeing. Touching and receiving touch are also both linked to delayed benefits in early child development^12,13^ and in adulthood^14^. For example, there is a causal effect of touch in promoting infant growth, emotion regulation, and motor development among others. Furthermore, skin-to-skin contact has been shown to help preterm infants gain body weight^15^ and in adulthood, interpersonal touch has been shown to reduce feelings of fear and stress^16,17^ as well as social exclusion^5^.

Overall, gentle touch and physical contact is generally perceived as pleasant and comforting in non-clinical samples^18–20^. Research has revealed that gentle touch is evaluated as pleasant regardless of the age of the touch receiver^19,21^. As shown in the research conducted by Croy et al.^21^, gentle touch applied to the skin of children aged between 5 and 12 years was favoured and rated as more pleasant compared to non-gentle touch over the skin. Similarly, Jönsson et al.^22^ revealed that 2-month-old babies demonstrated a preference for gentle touch applied to the forearm compared to CT non-optimal touch. Moreover, Ackerley et al.^18^’ study involved applying soft brush stroking to 5 body locations of adult participants: ithe forehead, arm, palm, thigh, and shin. Greater pleasantness ratings were provided when gentle skin stroking was applied,compared to CT non-optimal touch, which was evident for all body sites. Henceforth, whilst all these studies demonstrate that touch is experienced as pleasant when applied to various body regions in healthy populations, this is not always the case for individuals suffering from several psychiatric conditions, including for example, post-traumatic stress disorder^23^ and anorexia nervosa (AN), as well as individuals with Autism Spectrum Conditions^24–30^. Particularly in AN, a severe psychiatric condition characterised by profound body image disturbances (BIDs), including body size overestimations^31–33^, intense fear of gaining weight, atypical eating behaviour^34^, as well as impaired interoceptive awareness^35–42^, research has shown that individuals with acute or recovered AN rate interpersonal gentle touch as less pleasant overall, likely because of a generalized altered perception of tactile stimuli, which goes beyond their affective nature^24–30,43^. Furthermore, atypical social touch perception is accompanied by greater touch avoidance and social withdrawal^44–46^.

Nevertheless, all these studies suffer from important limitations. Firstly, the delivery of touch, whether vicariously or by means of a brush and/or by an (unfamiliar) researcher, although pleasant, do not resemble the affective, bodily interactions one encounters in real-life. In fact, social touch is an experience typically involving socially significant people, which in turn can substantially strengthen emotional bonds between them. These bonds are not only between the typically described dyad of caregiver and child, but also include romantic bonds and friendships. Importantly, the type of touch shared between two individuals is largely dependent upon the strength of the emotional bond^47–50^. Moreover, the area of the body that specific individuals are allowed to touch, is dependent upon the relationship and the identity of the touch provider, with romantic partners being allowed to touch intimate, private areas such as genitals, with touch across the whole body being evaluated as pleasant. In contrast, social affective touch from a stranger is largely evaluated as being unpleasant and is restricted to areas such as the hands^47,49,51–55^ (see^56^ for a comprehensive review). Using 2D human body outlines, Suvilehto et al.^47^ instructed participants to specify the body sites individuals of varying relationships to the participant (e.g., partner, parent, and stranger) were allowed to touch. Individuals identified as having closer emotional bonds, such as a partner or parent were able to touch more intimate body regions compared to touch from a stranger, which was restricted to the hands and upper torso only. Partners were the only individuals able to touch ‘taboo’ regions, such as the genitals and buttocks. Those closer to the touch receiver were able to touch more regions and these regions were classified as being pleasant to be touched^47^.

## The current study

Here, we took advantage of an imagined social touch interaction procedure by specifically manipulating the identity of the person delivering the touch to recreate more plausible scenarios of real-life affective tactile interactions. We also sought to establish whether imagined responses to social touch, which as reported by Suvilehto and colleagues^47^ are relationship-specific and ‘linearly dependent’ upon the body regions one receives touch to, also extend to individuals reporting varying levels of BIDs. Specifically, we focused on healthy individuals with low and high levels of BIDs, as research on subclinical populations also has the advantage of indicating whether somatosensory disturbances anticipate the onset of AN, therefore giving a direction for preventive measures. By doing so, this research will provide a basis for future research focusing on clinical populations, including AN, in order to understand the links with the pathology associated with eating disorder and social touch responses.

To this aim, and to address the limitations of correlational designs employed by previous studies^47^, we used a novel mobile application i.e., ‘*Virtual Touch Toolkit*’^57^ whereby individuals were instructed to interact with a 3D avatar of a human silhouette creating heatmaps indicating regions of the body they find soothing or unpleasant to be touched. Different touch interaction contexts can be selected from a menu. In the present study, participants created touch heatmaps for touch from ‘a loved one’ compared to an ‘acquaintance’. To identify individuals with low and high BIDs, participants also completed standardised self-reports of eating disorder symptomatology. Recent findings in healthy populations also revealed that lower levels of interoceptive awareness and greater levels of dysmorphic concerns are associated with lower preference for CT optimal touch (compared to CT non-optimal touch) when given by an experimenter (stranger)^58^. Accordingly, the contribution of body dysmorphic concerns, which commonly manifest in the general population, specifically in women^59,60^, and of interoceptive awareness towards soothing/unpleasant evaluations of social touch to socially acceptable body regions, was also measured using standardized self-report questionnaires.

Recent findings have shown that the closer the affective relationship between two individuals, the more social touching they are willing to accept from each other and the more pleasant they experience each other’s touch^47^. Accordingly, we expected to replicate the findings of this relationship-specific social touch, such that individuals would rate touch received from a loved one as more soothing than touch received by an acquaintance. Crucially, we seek to extend these results by looking at the effect of social relationship on touch pleasantness, depending on where the touch is delivered, that is on socially (un)acceptable body regions and in individuals reporting varying levels of BIDs. Based on recent findings with (healthy) populations that a reduced preference for social touch (from a stranger experimenter) is linearly associated with lower interoceptive awareness and higher dysmorphic concerns^58^, we expected that overall levels of unpleasantness to imagined social touch, whether from a loved one or an acquaintance, would be associated with lower interoceptive awareness and higher dysmorphic concerns.

## Results

### Demographics and self-report scales

Table 1 presents the means and standard deviations for participants’ age and questionnaire subscales, which have been calculated separately for high levels of BIDs and low levels of BIDs groups. The third column shows the result of pairwise comparisons between the two groups (Bonferroni-corrected). Both groups were matched for age. As expected, both groups differed regarding EDI-3^61^ subscale scores, with the high levels of BIDs group having significantly higher drive for thinness, greater body dissatisfaction, emotional dysregulation, interoceptive deficits, lower self-esteem, higher interpersonal alienation, ascetism (abstinence from sensual pleasure), maturity fears, more feelings of personal alienation and higher interpersonal insecurity compared to the low levels of BIDs group. As anticipated, both groups differed significantly in their EDRC score, with the low levels of BIDs group being representative of a healthy population (score < 18) and the high levels of BIDs group demonstrating a clinical group score, with the mean score greater than the cut-off point (score > 32). The two groups did not differ in their levels of perfectionism nor in their levels of dysmorphic concerns as measured by the DCQ (see Table 1).

**Table 1:** Mean and standard deviation (in brackets) of demographics and self-report questionnaires scores for the low levels of BIDs group (*n* = 35) compared to the high levels of BIDs group (*n* = 34).

	Low levels of BIDs (*n* = 35)	High levels of BIDs (*n* = 34)	Low vs. high level of BIDs
Age	25.77 (6.01)	30.68 (14.55)	t(67) = − 1.84, *p* = 0.07
EDI-3			
Drive for thinness	4.11 (4.29)	16.62 (6.05)	t(67) = − 9.93, *p* < 0.001
Body dissatisfaction	10.06 (6.76)	25.21 (9.15)	t(67) = − 7.83, *p* < 0.001
Bulimia	4.57 (4.84)	21.09 (8.82)	t(67) = − 9.68, *p* < 0.001
Low self esteem	4.40 (5.17)	15.32 (3.78)	t(67) = − 9.99, *p* < 0.001
Interpersonal alienation	6.20 (4.28)	14.21 (6.58)	t(67) = − 6.01, *p* < 0.001
Emotional dysregulation	5.14 (6.09)	16.32 (9.32)	t(67) = − 5.92, *p* < 0.001
Perfectionism	9.80 (5.02)	10.71 (5.06)	t(67) = − 0.75, *p* = 0.458
Ascetism	5.20 (4.67)	15.82 (7.84)	t(67) = − 6.86, *p* < 0.001
Maturity fear	9.46 (7.26)	15.15 (6.22)	t(67) = − 3.49, *p* = 0.001
Personal alienation	5.83 (4.42)	16.68 (6.90)	t(67) = − 7.8, *p* < 0.001
Interpersonal insecurity	7.71 (4.91)	14.97 (5.86)	t(67) = − 5.58, *p* < 0.001
Interoceptive deficit	6.83 (6.15)	19.21 (10.6)	t(67) = − 5.95, *p* < 0.001
EDRC	18.74 (10.35)	62.91 (16.18)	t(67) = − 13.55, *p* < 0.001
DCQ	14.80 (6.34)	14.32 (5.43)	t(67) = 0.33, *p* = 0.739

*EDI-3* eating disorder inventory 3; *EDRC* eating disorder risk composite*; DCQ* dysmorphic concern questionnaire.

### Main analyses

The social touch ratings are defined as positive/negative values from a scale ranging from (+ 100) soothing to (− 100) unpleasant. For the reporting of results, all means, and standard deviations are reported in brackets.

#### Imagined social touch ratings: individual bodily regions

The mean soothing/unpleasant ratings for all 13 bodily regions when touched by a loved one as compared to an acquaintance and for the two groups of low and high levels of BIDs respectively, are reported in Table 2.

**Table 2:** Mean (standard error of the mean in brackets) of soothing/unpleasant ratings for the implicit task for the two groups of Low (n = 35) and high levels of BIDs (n = 34). Scores depict ratings of imagined touchability for 13 bodily regions, according to the social relationship between the touch receiver and the touch giver (loved one vs. an acquaintance).

	Low levels of BIDs (*n* = 35)		High levels of BIDs (*n* = 34)	
	Loved one	Acquaintance	Loved one	Acquaintance
Buttock	12.45 (± 8.56)	− 50.96 (± 6.72)	26.12 (± 9.71)	− 50.6 (± 7.48)
Face	35.85 (± 6.37)	− 38.58 (± 5.96)	40.55 (± 7.49)	− 25.44 (± 8.76)
Feet	9.94 (± 8.43)	− 41.06 (± 5.69)	18.05 (± 8.72)	− 36.07 (± 7.06)
Groin	0.57 (± 10)	− 58.78 (± 5.58)	29.95 (± 10.26)	− 54.09 (± 6.96)
Hand	57.70 (± 6.05)	1.33 (± 6.67)	55.16 (± 6.05)	30.1 (± 8.01)
Lower arm	52.27 (± 6.15)	− 0.62 (± 7.23)	55.02 (± 6.17)	22.06 (± 8.24)
Lower leg	29.72 (± 7.31)	− 38.40 (± 7.08)	34.48 (± 7.71)	− 36.1 (± 8.25)
Shoulders and upper back	47.88 (± 6.35)	− 17.29 (± 6.13)	41.62 (± 7.86)	− 17.25 (± 8.9)
Shoulders and upper torso	23.92 (± 8.22)	− 46.03 (± 6.04)	39.53 (± 7.91)	− 44.89 (± 7.47)
Torso	15.94 (± 8.45)	− 62.07 (± 5.46)	21.23 (± 10.72)	− 55.54 (± 7.5)
Upper arm	48.38 (± 5.49)	− 1.40 (± 5.83)	49.53 (± 6.72)	10.59 (± 8.6)
Upper leg	22.54 (± 7.56)	− 51.28 (± 5.95)	35.15 (± 8.27)	− 50.71 (± 7.03)
Head	43.09 (± 5.90)	− 37.06 (± 5.53)	37.75 (± 6.84)	− 19.84 (± 7.58)

**Figure 1:**
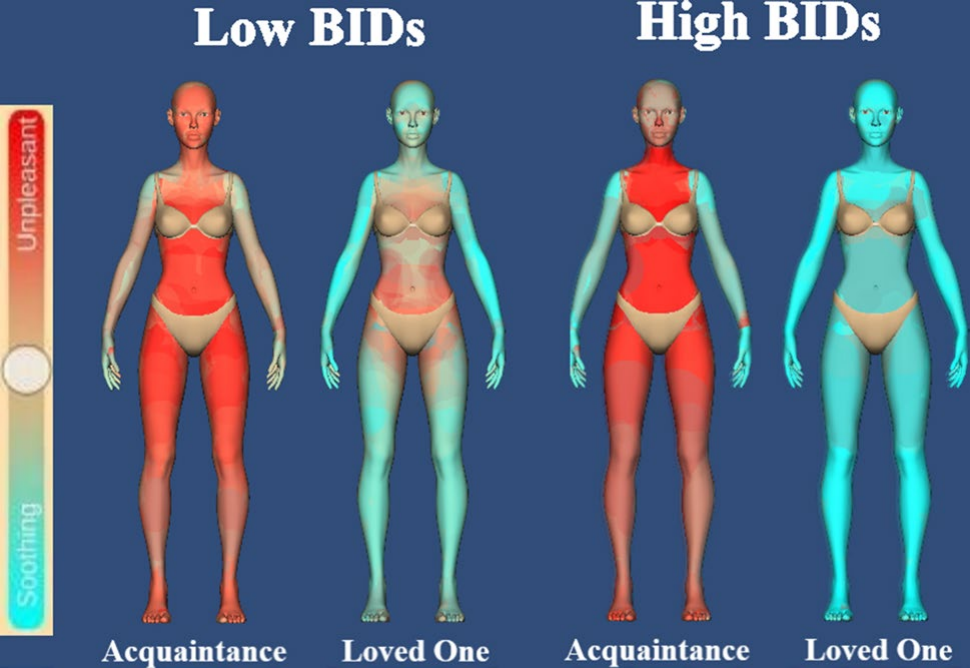
Relationship-specific soothing/unpleasant ratings for individual body zones, for the two groups of low vs. high levels of body image disturbances (BIDs), respectively. The image represents a female silhouette, with the option of selecting a male silhouette. Blue-leaning colours represent more soothing (pleasant) touch, whilst red-leaning colours represent less pleasant touch.

From a visual inspection of the relation-specific full-body avatars maps (see Fig. 1), both high and low levels of BIDs groups rated touch from a loved one as soothing regardless of the body zone. Body zones such as the abdomen and groin were rated as slightly less soothing compared to body zones such as the arm and hands. For an acquaintance, the abdomen was rated as the most unpleasant for both high and low levels of BIDs groups. Similarly, both groups rated the groin, upper leg, buttocks, shoulders/back, feet, legs, face, and head as unpleasant. The two groups differed in their ratings for the upper arm, lower arm, and hands, with the high levels of BIDs group rating these regions as more soothing compared to the low levels of BIDs group, who demonstrated more unpleasantness of touch from an acquaintance to these regions, except for the hands.

#### Imagined social touch ratings: intimate versus social body regions

##### Low levels of BIDs group

**Figure 2:**
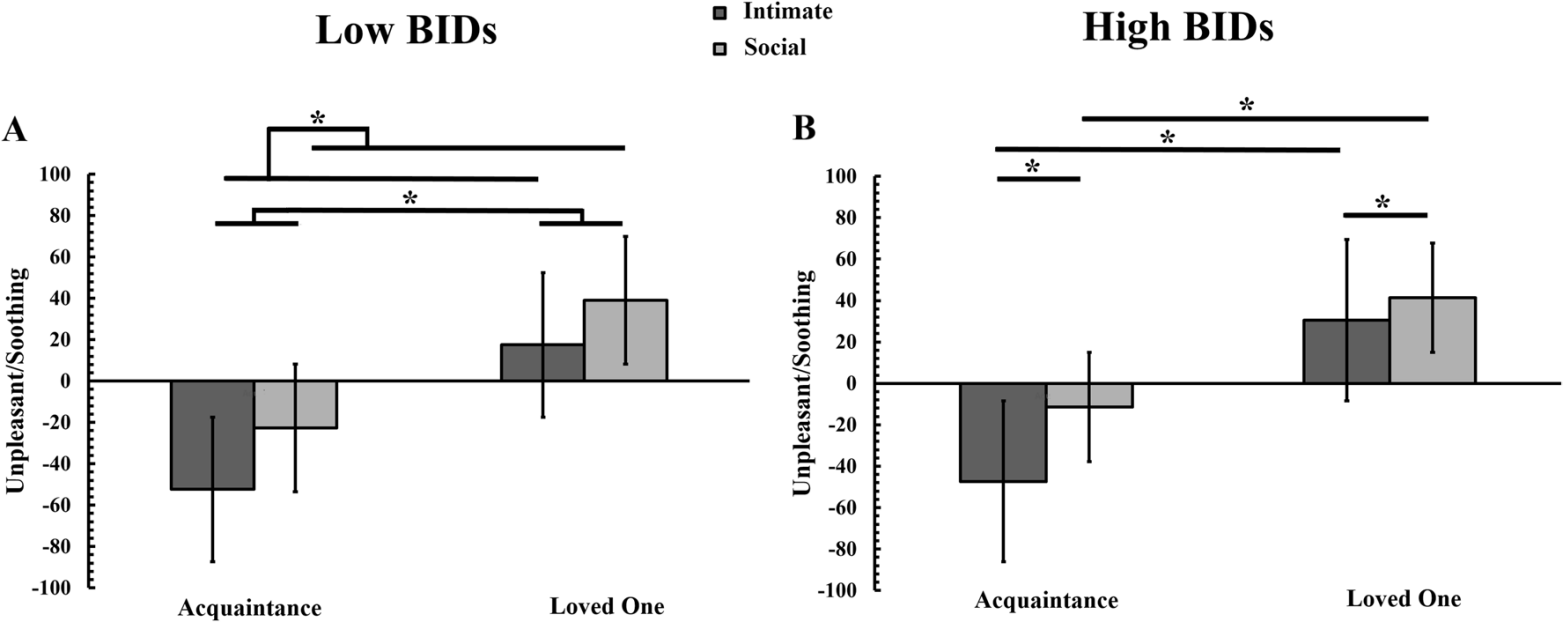
Mean (M) and standard error of the mean (S.E.M.) for soothing/unpleasant ratings for imagined touch delivered to each bodily regions (intimate vs. social) and for each relationship (acquaintance vs. loved one) for (**A**) low levels of body image disturbances (BIDs) group versus (**B**) high levels of BIDs group, respectively.

The 2-way ANOVA for the low levels of BIDs group revealed a significant main effect of Body Zone [*F*(1,34) = 56.557, *p* < 0.001, *ηp*^*2*^ = 0.625]. Social body zones were rated as significantly more soothing than intimate body zones which instead were rated unpleasant (8.27 ± 2.89 vs. − 17.43 ± 4.14, *p* < *0.0*01). There was also a significant main effect of relationship [*F*(1,34) = 71.262, *p* < . 001, *ηp*^*2*^ = 0.677], with touch from a loved one being rated as significantly more soothing than touch received from an acquaintance (28.29 ± 5.62 vs. − 37.45 ± 4.28, *p* < *0.0*01). However, the 2-way interaction of body zone × relationship was not significant [*F*(1,34) = 3.199, *p* = 0.083, *ηp*^*2*^ = 0.086], thus suggesting that low levels of BIDs group did not differ in their soothing ratings for touch to intimate and social body regions, depending on the social relationship with the touch giver. The lack of an interaction also suggests that social relationship and touch to bodily regions may mediate subjective pleasantness of imagined touch by at least partly independent mechanisms (see Fig. 2A).

##### High levels of BIDs group

The 2-way ANOVA for the high levels of BIDs group revealed a significant main effect of Body Zone [*F*(1,33) = 67.346, *p* < 0.001, *ηp*^*2*^ = 0.671]. Social body zones were rated as significantly more soothing than intimate body zones, which instead were rated as significantly more unpleasant (14.98 ± 5.38 vs. − 8.34 ± 5.84, *p* < *0.0*01). There was also a significant main effect of Relationship [*F*(1, 33) = 68.648, *p* < . 001, *ηp*^*2*^ = 0.675], with touch from a loved one being rated as significantly more soothing than touch received from an acquaintance (36 ± 7.16 vs. − 29.35 ± 6.23, *p* < *0.0*01). Importantly, these two main effects were further qualified by a significant 2-way interaction of body zone × relationship [*F*(1, 33) = 22.103, *p* < 0.001, *ηp*^*2*^ = 0.401]. Post-hoc comparisons revealed that participants displaying high levels of BIDs rated touch to intimate regions from a loved one as significantly more soothing than touch received by an acquaintance, which in turn was rated as unpleasant (30.60 ± 8.26 vs. − 47.28 ± 6.62, *p* < 0.001). Touch to social body regions was rated as significantly more soothing when received from a loved one as compared to touch received by an acquaintance, which in turn was rated as unpleasant (41.39 ± 6.41 vs. − 11.43 ± 6.49, *p* < 0.001, see Fig. 2B).

In summary, overall imagined touch was perceived as the most soothing when the individual giving the touch was familiar to the touch receiver i.e., a loved one, as compared to an individual less familiar to the touch giver, such as an acquaintance. Touch to intimate regions was perceived as the most unpleasant as opposed to touch to social regions. However, as demonstrated by the significant 2-way interaction, the effect of social relationship was dependent upon ‘where’ the touch was received, thus suggesting that information about the identity of the touch giver is pivotal in determining bodily maps of socially acceptable touch in participants demonstrating high levels of BIDs.

### Multiple regression analyses

By means of a series of multiple regression analyses, we assessed whether individuals’ differences in symptoms of dysmorphic concerns (DCQ) and of interoceptive awareness (interoceptive deficit EDI-3) could predict participants’ responses to imagined touch delivered to intimate/social body regions and depending on the social relationship with the toucher. To this aim, we ran linear multiple regressions to explore whether DCQ and interoceptive awareness scores were significant predictors of ‘Touchability Index’ (TI) for intimate body regions when touched by a loved one compared to a stranger, for the whole sample but also separately in the two groups of BIDs.

The linear multiple regression analysis calculated to predict TI for intimate body regions touched by a loved one from symptoms of dysmorphic concerns and of interoceptive awareness for the whole sample was significant [*F*_(2,66)_ = 3.456, *p* = 0.037, *R*^2^ = 0.308], with interoceptive awareness emerging as the only significant predictor (*p* = 0.023). However, this finding seems to be specifically driven by the high levels of BIDs group. The linear multiple regression analysis calculated to predict TI for intimate body regions touched by a loved one from symptoms of dysmorphic concerns and interoceptive awareness was significant in the high Levels of BID group [*F*_(2,31)_ = 4.199, *p* = 0.024, *R*^*2*^ = 0.462], with interoceptive awareness emerging as the only marginally significant predictor (see Table 3). The regression equation for social body regions touched by a loved one only approached significance, with interoceptive awareness being a marginally significant predictor [*F*_(2,31)_ = 3.148, *p* = 0.057, *R*^*2*^ = 0.411, see Table 3]. Similarly, no significant regression equations were found for any of the TI conditions (intimate and social regions) with dysmorphic concerns and interoceptive awareness in the whole sample or separately for the low levels of BIDs group. In summary, for participants displaying high levels of BIDs, (greater difficulties in) interoceptive awareness was associated with higher soothing ratings for intimate and social body regions when touched by a loved one.

**Table 3:** Unstandardized coefficients from the linear multiple regression models of dysmorphic concerns and emotional awareness predictors of the touchabiliy at intimate and social bodily regions when touched by a loved one versus an acquaintance, respectively.

Touchability										
	Low levels of BIDs Group (n = 35)					High levels of BIDs Group (n = 34)				
	B	SE	*β*	*t*-value	*p*-level	B	SE	*β*	*t*-value	*p*-level
Intimate bodily regions touched by a loved one										
Interoceptive deficit (EDI-3)	− 0.01	0.19	− 0.07	− 0.06	0.95	0.31	0.16	1.43	1.96	0.06
DCQ	0.06	0.19	0.39	0.32	0.75	− 0.30	0.16	− 2.70	− 1.80	0.07
Social bodily regions touched by a loved one										
Interoceptive deficit (EDI-3)	0.09	0.18	0.45	0.50	0.62	0.32	0.16	1.11	1.92	0.06
DCQ	0.13	0.18	0.64	0.74	0.47	− 0.23	0.16	− 1.57	− 1.39	0.18
Intimate bodily regions touched by an acquaintance										
Interoceptive deficit (EDI-3)	0.06	0.18	0.30	0.34	0.74	0.16	0.17	0.58	0.91	0.37
DCQ	0.18	0.18	0.86	1.01	0.32	− 0.18	0.17	− 1.30	− 1.05	0.30
Social bodily regions touched by an acquaintance										
Interoceptive deficit (EDI-3)	0.23	0.18	0.93	1.32	0.20	0.11	0.18	0.39	0.62	0.54
DCQ	0.09	0.18	0.35	0.51	0.61	− 0.12	0.18	− 0.81	− 0.65	0.52

*EDI-3* eating disorder inventory-3; *DCQ* dysmorphic concern questionnaire.

## Discussion

By using a factorial design, this research is the first to systematically investigate soothing and unpleasant ratings to imagined social touch when delivered to intimate body regions by a loved one (vs. a distant acquaintance), specifically in individuals displaying varying levels of BIDs. To achieve this, the *Virtual Touch Toolkit*, an interactive media mobile application for promoting wellbeing through social touch^57^ was employed to quantify relationship-specific maps of bodily regions ‘where’ social touch is perceived is pleasant, depending on the social relationship with the touch giver (‘who’). Furthermore, we wanted to understand the contributions of individual differences in appearance-related dysmorphic concerns and interoceptive awareness difficulties, i.e., two key traits associated with the risk, maintenance, and illness symptomatology of AN^62,63^, to relationship-specific differences in where on the body social touch is rated as pleasant.

Overall and as anticipated, results demonstrated that touch from a loved one (a romantic partner) is evaluated as more soothing compared to touch from an unfamiliar person i.e., an acquaintance from whom the touch was instead perceived as highly unpleasant. These findings corroborate evidence obtained by previous studies using similar approaches. For instance, research by Suvilehto et al.^47–49^ shows that the social relationship shared between the touch giver and the receiver influences perceived pleasantness of social touch, indicating that relationship-specific social touch is closely related to the maintenance and establishment of social bonds in nonhuman primates^64^ but also in humans. If the touch giver is closer and more familiar to the touch receiver, the more soothing this touch is perceived and the more areas are evaluated as pleasant^47,48,53–55^. On the contrary, if the touch is given by a stranger, then this is largely perceived as unpleasant, with the body regions that an individual can be touched being more restricted^47^. Our study extends these findings by examining relationship and body site specific differences as a function of individual differences in interoceptive awareness and dysmorphic concerns.

For individuals reporting low levels of BIDs, independently from the bodily regions one is allowed to be touched, we found that, on average, imagined touch from a loved one was rated as significantly more soothing than touch received from an acquaintance. Furthermore, imagined touch to social body zones was rated as significantly more soothing than intimate body zones, which instead was rated unpleasant. Yet, the interaction between social relationship and touch to socially acceptable bodily regions was not significant, which suggests the two factors may mediate subjective soothing of imagined touch by at least partly independent mechanisms, and therefore, prompting for a social relationship tuning and bodily regions’ independence of social touch mechanisms. These findings may seem partially in contrast with those reported by Suvilehto and colleagues^47–49^ according to which the social relationship shared between the touch giver and the touch receiver intimately influences perceived pleasantness of social touch, with the bodily areas where touching is allowed being ‘linearly dependent’ on the emotional bond with the toucher^64^. Nonetheless, we may point to some dissimilarities between Suvilehto et al.’ investigation^47^ and our study which pertain to methodological differences. For instance, in the study by Suvilehto et al.^47^, first the reasons for social touching across different social relationships was explored (for e.g., “greeting” or “comforting”). Then, participants reported when they had last seen each network member. The authors found that emotionally closer individuals in inner layers of the social network were allowed to touch wider bodily areas for more reasons. However, frequency of social contact with an individual did not predict the areas acceptable for social touch. Taken together these results provide further evidence to the relatively extensive literature of the effects of contextual factors on social touch, according to which the experience of social touch is strongly modified by a variety of toucher-related and situational factors, including relationship status and group membership (see Saarinen et al.^56^ for a review). Furthermore, it should be noted that family members may use touch to express a wider array of emotions via touch than strangers. Strangers commonly convey universal or prosocial emotions via touch whereas family members express also social control and negative affective states such as pride, envy, or psychological control or dominance, punishment, farewell, hurting, or scaring^47,65,66^. Following this reasoning, it might be possible that in the case of our study, individuals’ understanding of the communicative intentions (i.e., emotions or others’ mental states) of the tactile interaction might have been hindered somehow by the lack of any relevant contextual cues, thus in turn modulating the hedonic processing of touch when received by a meaningful person and specifically to socially acceptable bodily regions. Nonetheless, our explanation is not conclusive and future studies using the *Virtual Touch App* should focus on probing social touch responses whilst carefully looking at the meaning and exposure to social touch occurs.

Two competing hypotheses can be formulated for the imagined social touch experience in individuals displaying high levels of BIDs. One possibility in keeping with previous investigations focusing on AN patients which revealed that the more severe the BIDs, the more uncomfortable and less likely individuals with AN engage with physical intimacy^67^, was that individuals reporting high levels of BIDs would find touch unpleasant regardless of the social relevance of the touch giver and at all bodily regions. This was not the case for the present study. On the contrary, individuals displaying high levels of BIDs evaluated imagined touch from a loved one at social body regions as the most soothing touch, compared to all other conditions. Furthermore, unpleasantness ratings were specifically evident when touch was imagined from an acquaintance and when it was delivered to an intimate body region. Therefore, in line with the second hypothesis and as demonstrated by the significant interaction of social relationship and bodily regions, here, we show that bodily regions are rated as soothing strictly depending on the social relationship with the touch giver, thus suggesting that subjective pleasantness of imagined touch may stem from dependent mechanisms. Therefore, our findings may align with the results obtained by the study of Tagini and colleagues^29^ which demonstrates a ’preserved’ experience of imagined affective touch (although by a stranger) in women with AN when using an imaginary social touch procedure. However, it should be noted that a crucial limitation of Tagini et al.’s investigation^29^ is the lack of differentiation between imaginary affective touch delivered by a loved one, compared to a stranger. The ’preserved’ experience of imagined affective touch when delivered by a stranger in women with AN in their research setting may not be comparable to the experimental protocol adopted in our study. Moreover, it might not be sufficiently veridical of real-life scenarios where tactile interactions normally involve meaningful people.

One additional point of novelty of our study is that in individuals displaying high levels of BIDs (but also in the whole sample), higher levels of interoceptive awareness, i.e., greater difficulties with being aware of the connection between body sensations and emotional states, were associated with greater soothing evaluations of imagined touch when delivered by a significant individual and specifically to intimate bodily regions (although it should be noted that statistical results for social bodily regions reveal a similar trend). Recent research has shown that affective touch is crucial for the healthy development of infants but also for the emotional regulation of adults^2^. In older infants, it has been shown that in addition to self-soothing behaviours like thumb sucking, infant’s physiological arousal can be regulated by soothing, touch-based behaviours by their caregivers^68,69^. Accordingly, positive interpersonal tactile interactions seem able to “bind” together inner feelings about the state of the body with external perceptions of the body and the world^70^.

On the other hand, it is known that individuals suffering from AN experience interoceptive difficulty and affective regulation (including alexithymia and poor emotional clarity)^71^. Here, we complement the research in clinical populations by extending the results of the key role of interoceptive awareness problems in meaningful affective tactile interactions to individuals displaying high levels of BIDs. Consistent with the idea of the role of interoceptive deficits in maintaining EDs symptomatology^72–74^, problems with interoceptive awareness may play a role in maintaining psychopathology by lack of recognition of the severity of the illness and homeostasis^75^. Accordingly, we may speculate that sensitivity to relationship-specific bodily patterns of social touch in individuals with high levels of BIDs might be mediated by greater problems with interoceptive awareness, which in turn may play a role in the experience of bodily affective contacts, a hypothesis which warrants further investigation. Nevertheless, our interpretation of the results may not entirely explain the direction of the association between (greater) interoceptive awareness problems and (higher) soothing responses to social touch by a meaningful person, and further exploration of the factors which might have mediated this relationship are needed. For example, a suitable candidate for future research explorations would be (insecure) attachment style. Interoceptive awareness and the accurate assessment of one's interoceptive states has been thought to develop through early attachment experiences^76^. For instance, in the case of anxious attachment style, a fear of abandonment and rejection yet desire for closeness from significant others is observed^77^. A recent study by Ferraro and Taylor^78^ also reported that interpersonal relationships distinguished by either an under or over reliance on others for comfort and reassurance, are negatively associated with the conscious perception of internal states and understanding how these sensations might indicate emotional states. A further study by Krahe and colleagues^79^ looking at the sensitivity to CT-optimal touch and adult attachment styles, reported that higher scores in attachment anxiety were associated with reduced pleasantness discrimination of CT-optimal (compared to non-CT optimal touch). On the other hand, attachment style was not related to cardiac (interoceptive) accuracy as measured by the heartbeat perception task^80^, an interoceptive measure profoundly different from the one used in our study. Accordingly, the results we obtained in our study might be an ‘epiphenomenon’ of a particular (insecure) attachment style in our participants displaying high levels of BIDs. In future, it would be of interest for prospective research to account for the impact of interoceptive awareness difficulties on soothing touch responses by a caregiver by comparing individuals' attachement styles in clinical and non-clinical samples with EDs.

Nevertheless, it could be that individuals with high levels of BIDs might be better ‘attuned’ to the social relevance of the touch giver and the body sites a specific individual is allowed to touch, thus pointing to a (potentially) higher sensitivity to relation-specific bodily patterns of social touch. If this was proved to be the case, our results can provide useful hints for future research, but also in clinical settings, particularly for mindful awareness in body-oriented therapy (MABT). MABT is a mindfulness-based approach specifically designed to teach interoceptive awareness and related skills for self-care and emotion regulation. Specifically, MABT involves a focus on sensation guided by the use of ‘touch’ to support learning interoceptive awareness, for example by asking the client what is noticed in response to physical pressure on an area where there is expected sensation, like in the case of physical tension or apparent discomfort^81^.

In light of the observation that individuals with high levels of BIDs may have a greater sensitivity to touch, specifically depending on ‘who’ is touching them and ‘where’ they are touched, which in turn is associated with greater problems with interoceptive awareness, we can suggest at least two reasons as to why this information could prove useful in the clinical practice. One reason pertains to the ability of building ‘trust’ in the client/therapist relationship, which is one of the most important clinical elements for the client’s successful engagement in accessing interoceptive awareness. Secondly, our results could be useful for facilitating the client’s trust in their own body, by connecting to the body and the emotions they can feel safe. Accordingly, imaginary methods like the *Virtual Touch Toolkit* might be combined with body-oriented therapies to provide at the beginning of the therapeutic journey a ‘safe’ space where client and therapist may explore together feelings of interpersonal contacts during a proposed exercise. This way, the therapy could be modified so that touch to a proximal body area, away from a negatively experienced/unpleasant ‘targeted area’ for interoceptive awareness, is used instead to promote interoceptive engagement. Alternatively, client self-touch could be used in situations which, or by clinicians for whom, touch is perceived as not appropriate^81^. Ultimately, by promoting a positive experience of somatic wellbeing, the therapist can motivate a client to engage in further therapeutic work and can lead to further development of inner resources for daily life and increased emotion regulation.

## Limitations

Our findings should also be viewed considering the study’s limitations. Firstly, individuals who participated in our study were only at risk of BIDs and did not have a formal diagnosis of an eating disorder like AN. Given that our participants are not a clinical population, it might be the case that they may not struggle with maintaining relationships or do not display atypical responses to social touch like individuals with AN do. Future studies should concentrate on examining soothing experience of interpersonal contacts in clinical samples of women suffering from AN, so to provide more conclusive evidence on the role of social relationship in meaningful affective interactions. Also, further research focusing on social touch experiences with clinical and non-clinical samples should consider levels of exposure to positive touch and being in a relationship in which touch is given daily^10,82^. For instance, previous research has revealed that (healthy) individuals in satisfying relationships experience and rate touch as more pleasant than those in less satisfying relationships^10,82,83^. Accordingly, it could be that individual differences in the degree of exposure to touch, as well as who are in a relationship or married, experience greater exposure to touch and are more likely to tolerate and rate this touch as more soothing than those who are single^82^.

A caveat of our findings’ interpretation of a role of interoceptive awareness deficits in relationship-specific maps of bodily regions of social touch allowance, is that the conclusions drawn here, relate only to self-reported interoceptive deficits measured by the Interoceptive Awareness subscale of the EDI. For instance, the EDI has been criticised as an assessment of interoception, given that it primarily considers the emotional rather than its somatic aspect^84^ and it also fails to differentiate between a confusion or lack of clarity regarding internal experiences and non-acceptance of affective arousal^85^. Future research should use objective and subjective measures of interoception to overcome this limitation.

Furthermore, it is possible that gender differences might have played a role in this mechanism. With these regards, studies on gender differences in affective touch demonstrate that men are more confident in touching than women^86^ (see also^87^ for a review). A study by Trotter and colleagues^88^ which examined sex differences using the touch experiences and attitudes questionnaire has confirmed that overall women valued touch more positively than men except for touch towards strangers, which was more comfortable for men than for women^88^. More recently, Sorokowska and colleagues^89^ collected a large and heterogenous sample across 45 different countries, whereby participants indicated whether they had employed different touch actions (embrace, hug, kiss, stroke) towards different person groups (partner, child, female friend, male friend) in the last week. An affective touch diversity score derived from the sum of reported interactions was greater in women than in men. As for our study, preliminary analyses suggest this is not the case. Here, gender does not seem to modulate relationship-specific maps of bodily regions of social touch allowance, *depending* on the levels of BIDs. Indeed, whilst no gender difference was found for touch received to social areas, women rated touch to intimate areas as less pleasant than men did. These effects were comparable in the two levels of BIDs groups (see Supplemental materials for an additional analyses on the effect of gender on soothing/unpleasant ratings, depending on social relationship and body zones, Fig. S1). Nevertheless, we believe these results should be interpreted with caution given the small number of men, compared to women recruited in our samples, as well as within the two groups of BIDs. In future, the designation of touch zones by same versus opposite-gender of the touch giver through the proposed digital *Virtual touch toolkit* could shed more light on the aspect of societal gender norms and provide quantifiable evidence about touch boundaries, specifically in the case of individuals suffering from eating disorders.

Finally, touch deprivation during the Covid-19 pandemic could also have played a role in explaining findings for this study^90,91^. It should be noted that data collection for this study began during the Covid-19 pandemic and as such the unpleasantness towards touch from an acquaintance may be consequent to fear of touching another and social distancing to avoid infection, a fear instilled towards individuals unknown to us^92^. Accordingly, it could have been that fear and unpleasantness towards touch may have manifested post pandemic, altering how individuals respond to touch from someone less familiar^92^.

## Conclusions

Findings from this study corroborate previous results that soothing responses to imagined social touch are crucially dependent upon the social relationship shared between the touch giver and the touch receiver, as well as the bodily regions the touch giver is allowed to touch. Importantly, we add to the current literature by providing first causative (compared to correlational) evidence that in individuals with high levels of BIDs, ratings of soothing tactile experience crucially depend upon the interaction of ‘who’ is delivering the touch and ‘where’ on the body the touch giver is allowed to touch. Importantly, interoceptive awareness deficits might play a role in this mechanism by modulating this greater sensitivity to relationship-specific bodily patterns of social imagined touch in individuals with high levels of BIDs. Accordingly, it might be plausible that participants displaying high levels of BIDs may be better ‘attuned’ to the social relevance and the body regions a specific individual is allowed to touch, with greater interoceptive awareness deficits supporting this relation-specific bodily patterns of imagined social touch, a hypothesis which warrants further consideration in future studies.

Given the successful application of the *Virtual Touch Toolkit* for the assessment of individuals with high levels of BIDs’ responses to imagined social touch in the current study, future research might benefit from the use of this Toolkit as an intervention tool capable of probing the possible interaction between the interpersonal difficulties generally observed in AN individuals and the greater sensitivity to imagined experience of affective bodily contacts in relation to ‘who’ is touching them and ‘where’ they are touched. This could be achieved by incorporating methods of assessment of physiological and behavioural responses to imagined affective tactile interactions as measured by the *Virtual Touch Toolkit* whereby different social relationships to the touch receiver are probed in patients suffering from AN. In this regard, and beyond the well-known positive outcomes of touch-based body-oriented therapy, including massage in eating disorders^93,94^, qualitative research has also found that touch positively mediates health professionals’ interactions with patients^95^ by helping with building boundaries, expressing caring, and fostering emotional closeness. This is especially important in body-oriented psychotherapies, including MABT for AN^96^ because physical contact is fundamentally related to the therapeutic process. The *Virtual Touch Toolkit* provides a ‘virtual’, safe space which resembles real life scenarios in which a patient may gradually attend to their own body, but also, familiarize with the interpersonal contact received by a stranger. In future, the feasibility of imagined social touch interaction protocols of social touch based on the use of digital tools like the *Virtual Touch Toolkit* for rehabilitative purposes in AN should be tested.

## Methods

### Participants

A total sample of 69 participants (22 males and 47 females, M_age_ = 28.19 years, SD = 11.27, range: 18–48 years) were recruited. Participants were allocated to two groups of high levels of BIDs or low levels of BIDs, based on the median Eating Disorder Risk Composite (EDRC) score (med = 39, high levels of BIDs group: M = 62.91, SD = 16.18 & low levels of BIDs group: M = 18.74, SD = 10.35), obtained to the Eating Disorder Inventory (EDI-3) scale, which is a self-report questionnaire administered to assess eating disorder symptomatology^61^.

Accordingly, a total of 34 participants aged 20–66 (M_age_ = 30.68yrs, SD = 14.55), consisting of 12 males and 22 females were assigned to the high levels of BIDs group. An additional 35 participants aged 18-41yrs (M_age_ = 25.77, SD = 6.01), comprising of 10 who were male and 25 who were female were assigned to the low levels of BIDs group. See Table 1 below for a breakdown of EDI-3, DCQ scores and age information for the two groups. The total sample size for this study was calculated through a G-Power 3.0.10 power analysis^97^, which indicated a minimum sample of 60 participants was required for a medium effect (f = 0.25) with 95% power, using a Mixed ANOVA with alpha at 0.05 (two tailed).

Participants for both the high and low levels of BIDs groups were recruited from the UK and in Germany through various platforms such as social media, recruitment agencies, external contacts, and the Liverpool John Moores University SONA participant recruitment scheme. All participants self-reported not to possess any psychiatric or neurological disorders (including a current diagnosis or previous eating disorder formal diagnosis). All participants had normal or corrected vision, did not have any form of skin or chronic pain conditions such as eczema or fibromyalgia and were not pregnant.

All participants gave informed consent to participate and at the end of the study were provided with a full debrief and overview of what the study was about. Participants were offered £5 shopping vouchers as a compensation for their time. Level 4 BSc Psychology students received SONA credits. The study was carried out in accordance with the Helsinki declaration of ethical standards. The study protocol was approved by Liverpool John Moores University (LJMU)’s University Research Ethics Committee (UREC, protocol: 21/PSY/013).

### Measures

#### The virtual touch toolkit

The virtual touch toolkit is a new interactive media mobile application which can be downloaded by users to their mobile (android or iOS) or tablet (Android only) and freely interact through various exercises^57^. For this study, participants were provided with detailed instructions of how to download the application for both Android and iOS phone and how to navigate to the exercise “My body in your hands”. Participants were asked to create a user account by selecting their gender, age, and were asked to select either a male or female silhouette which represents the gender they identify with. To ensure confidentiality, no names or identifiable information was asked, instead participants entered a unique participant code which matched their app data with their questionnaire data.

**Figure 3:**
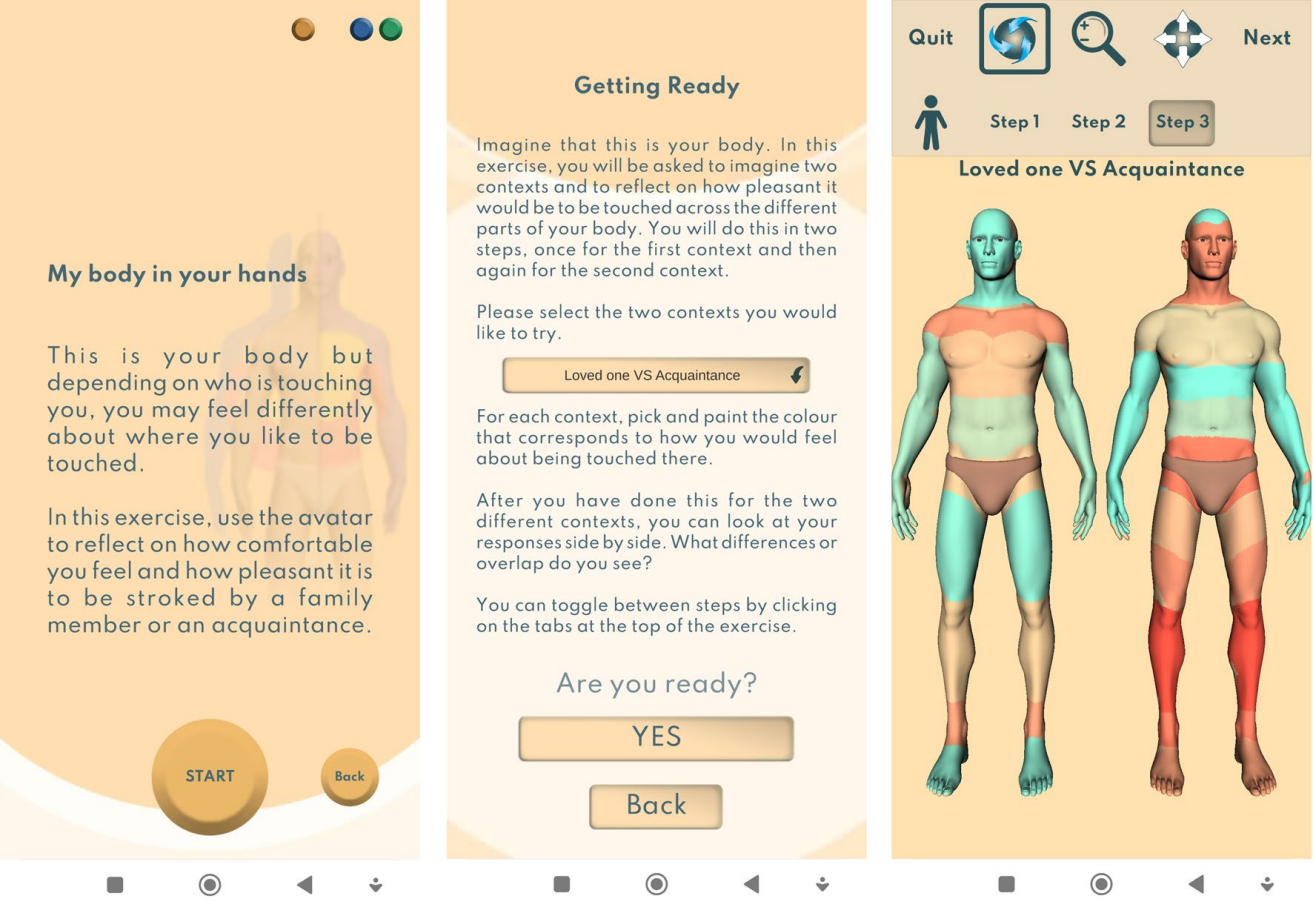
“My body in your hands” exercise. Using a user-defined avatar, participants were given the tools to indicate their perceived pleasantness (soothing) of social touch across intimate (e.g., groin) and socialbody parts (e.g., shoulder) when touched by a loved ones and an acquaintance, respectively. The image represents a male silhouette, with the option of selecting a female silhouette. Blue-leaning colours represent more soothing (pleasant) touch, whilst red-leaning colours represent less pleasant touch.

Participants engaged with the exercise titled: “My body in your hands”. For this exercise, participants interacted with a 3D virtual ‘human’ model and were instructed, using brush tool and a colour scale (blue = soothing (+ 100) to red = unpleasant (− 100)), to specify which body areas they evaluate as being soothing or unpleasant to be touched from a loved one compared to an acquaintance. Participants imagined being touched to various bodily regions from a loved one and an acquaintance and were instructed to demonstrate what regions they would find soothing and unpleasant to be touched (see Fig. 1). To make certain that an accurate average of the body region could be taken, participants were instructed to colour in the entire surface of the avatar and could use the zoom in tool to reach smaller regions such as the fingers. To ensure consistency, for the ‘loved one’ scenario, participants were asked to imagine being touched by a romantic partner. Two separates individual heatmaps for both touch providers were created i.e., a one for a loved one and one for an acquaintance. Prior to submitting their responses, participants were presented with a final step where they could see both avatars for loved one versus acquaintance displayed together, to ensure they were happy with their responses, and they were correct for each condition. Participants could manipulate the size of the brush tool, which prevented any cross contamination of colour to different regions. Overall, this task took participants approximately 10 min to complete (see Fig. 3).

#### Self-report questionnaires

##### The eating disorder inventory-3 questionnaire

The Eating Disorder Inventory-3 (EDI-3)^61^ is a 91 item self-report questionnaire assessing eating disorder symptomatology. This questionnaire assesses 12 subscales, 3 of which assess eating disorder symptomatology. A total of 6 subscales are calculated: the eating disorder risk (EDCR), ineffectiveness, interpersonal problems, affective problems, over control and general psychological maladjustment. This questionnaire was administered to examine participants’ risk of EDs (high vs. low levels of BIDs). The EDRC score which is calculated based on 3 of the subscales: body dissatisfaction, drive for thinness and Bulimia was used to assign a participant to one of two groups: high levels of BIDs versus low levels of BIDs group, based on a median split procedure. Typically for this questionnaire a cut off of 18 or below is indicative of no eating disorder risk and a score of 32 or above demonstrates high eating disorder risk. Following a previous study by our group^58^, we focused on the subscale ‘Interoceptive Deficits’ as a measure of 'interoceptive awareness’ to explore the relationships between subjective experience of social touch at different body sites and when received by a loved one compared to a stranger, with self-reports of interoceptive awareness^61^. The EDI-3 was validated in clinical and non‐clinical samples and has shown good internal consistency (α = between 0.75 and 0.92 for each subscale), and excellent sensitivity and specificity^98^. This questionnaire has largely been used previously with non-clinical^99,100^ and subclinical populations^58^ and has shown to be successful in identifying individuals at risk of an EDs^100^.

##### Dysmorphic concern questionnaire

The dysmorphic concern questionnaire (DCQ)^101^ is a short, 7-item questionnaire used to measure an individual’s concern towards their physical appearance. Items focus on the belief of being misshapen or malformed despite others’ opinion; belief in bodily malfunction (e.g., malodour); consultation with cosmetic specialists; spending excessive time worrying about appearance; and spending a lot of time covering up perceived defects in appearance. Participants were asked to rate each item on a Likert scale from a minimum of 0 (“not at all”) to a maximum of 4 (“much more than most people”). Total scores range from 0 to 28 with a critical value of 9 usually indicating clinical concern^99^. It should be noted that, compared to traditional scales measuring EDs risk (for example, the eating disorder examination-questionnaire^102,103^), the DCQ measure different sets of psychopathological features at least in university student populations^104^. Therefore, this scale was included to determine whether higher levels of dysmorphic concerns (separately from EDs symptomatology as measured by the EDI-3) were associated with greater unpleasantness to social touch at different body sites and when received by a loved one, compared to a stranger. The DCQ has been shown good internal consistency with α = 0.80^101^.

### General procedure

This study was conducted both online and, in the lab, and used Qualtrics software, Version 60,939 of the Qualtrics Research Suite (Copyright © 2015 Qualtrics., Provo, UT, USA. http://www.qualtrics.com). This way 53 participants completed the study online and 16 participants completed the study in the lab. Importantly, both versions of the study used the same weblink to keep it consistent. Due to Covid-19 restrictions, we decided it was necessary to move from online to lab-based data collection, this was due to the lack of engagement from participants and the difficulties when downloading the application to their mobile device. Also, during the beginning of the study, restrictions were still in place meaning that participants could not take part to face-to-face research, which was another reasoning behind the original online data collection. Participants were emailed with an invitation which comprised of a brief overview of the study. Those that took part online were provided with a blink to the study and were instructed to click the hyperlink if they were happy to participate. All participants received an electronic version of the information sheet and were asked to provide consent to take part. After providing consent to take part, participants were given a unique code generated randomly through Qualtrics and asked to take note of this. Participants used this unique code on all questionnaires and on the task. This ensured that their Qualtrics questionnaire responses could be linked to their task data collected on the Virtual touch app. Participants then were given a detailed instruction of how to download the app, how to create their user profile using their unique code and where the task was located on the app. Proceeding from downloading the app, participants completed the “my body in your hands” exercise by completing Heatmaps demonstrating the regions of the body the find soothing and unpleasant to be touched from a loved one compared to an acquaintance using a colour scale (blue = soothing (+ 100) to red = unpleasant (− 100)) and brush tool. After completion of the task, participants filled out a series of questionnaires through Qualtrics. These questionnaires measured body image disturbances, body image concerns, body image misperception and body awareness through the EDI-3^61^ and DCQ^101^. The order of administration of these questionnaires were fully counterbalanced for each participant. Overall, this study took both online and lab participation 35 min to complete. This study began on 3rd August 2021 and ended on 31st March 2022.

### Data handling

Data were analysed using IBM SPSS 26 (IBM Corp. Released 2020. IBM SPSS Statistics for Windows, Version 26.0. Armonk, NY: IBM Corp). Inspection of model residuals indicated data were normally distributed. Assumptions of sphericity were not violated. Based on findings from Suvilehto et al.^47^, we categorised the body zones: Abdomen, Groin, Buttocks, Upper Leg and Face as ‘intimate’ regions, given only more intimate exchanges involve touch to these areas. We grouped ‘social’ regions as the Head, Hands, Upper Arm, Lower Arm, Lower Legs, Feet, Back and Shoulders. As in Suvilehto et al.^48^, we computed a ‘Touchability Index’ (TI) which is the proportion of coloured pixels within the body outline for each touch area maps. This was calculated through averaging out the regions classified as intimate with the regions classified as social for each relationship (loved one and acquaintance). First, independent samples *t* tests (Bonferroni-corrected) were conducted to assess group differences for subscales of the EDI-3 and DCQ questionnaires, by examining significant differences in mean scores across each BIDs group. In the next step, we conducted a preliminary analysis according to which soothing/unpleasantness ratings of social touch were entered in a 3-way Mixed Analysis of Variance (ANOVA) with a between-subjects factor of Group (2 levels: high levels of BIDs and low levels of BIDs) and two within-subjects’ factors of Relationship (2 levels: loved one and acquaintance) and Body Zone (2 levels: intimate and social regions). These analyses showed that both low and high levels of BIDs groups provided higher soothing ratings of social touch for social than for intimate body regions [*F*(1, 67) = 120.946, *p* < 0.001, *ηp2* = 0.064] and for a loved one than for an acquaintance [*F*(1, 67) = 139.868, *p* < 0.001, *ηp*^2^ = 0.068]. The 2-way interaction of relationship × body zone was also significant [*F*(1, 67) = 22.549, *p* < 0.001, *ηp*^2^ = 0.025], and it was further qualified by a significant 3-way interaction of group × relationship × body zone [*F*(1, 67) = 5.874, *p* = 0.018, *ηp*^2^ = 0.08], showing different relationship × body zone effects according to the levels of BIDs group. Accordingly, our main analysis design aimed to detect whether the significant interaction of relationship × body zone was driven by any of the two groups and involved two separate 2-way ANOVAs with Relationship (2 levels: loved one and acquaintance) and Body Zone (2 levels: intimate and social regions) as within-subject variables in each level of BIDs group. Note that for the sake of completeness, we also report the mean Touchability (TI) scores for all 13 bodily regions separately for imagined touch received by a loved one, as opposed to an acquaintance and respectively for the low levels of BIDs and high levels of BIDs groups (see Table 3).

In the last step, we explored the associations between TI for intimate/social regions touched by a loved one compared to an acquaintance with individual scores of DCQ, and interoceptive awareness (Interoceptive Deficits, EDI-3). A series of multiple linear regressions were fit to predict the two self-report measures of body image disturbances for each soothing/unpleasant tactile evaluations, depending on the social relationship with the toucher and the (intimate vs. insensitive) bodily regions they were allowed to touch. All data are reported as Mean (M) and Standard Error of the Mean (S.E.M.). The source of all significant interactions was analysed using Duncan post hoc tests. This test has been developed to reduce the risk of false negative (Type II) error when correcting for multiple comparisons^105^, by reducing the size of the critical difference depending on the number of steps separating the ordered means. Using this procedure is deemed optimal for testing in the same design those effects that may have different sizes^106–108^. A significance threshold of *p* < 0.05 was set for all effects and effect sizes were estimated using the partial eta square measure (*ηp*^2^).

### Ethics approval

The study was carried out in accordance with the Helsinki declaration of ethical standards. The study protocol was approved by reviewed and approved by Ethics Commission of the Universität der Bundeswehr München (Ethikkommission UniBw M) and by the Liverpool John Moores University (LJMU)’s University Research Ethics Committee (UREC, protocol: 21/PSY/013). All participants provided their written informed consent to participate in this study.

### Consent to participate

Informed consent was obtained from all individual participants included in the study.

### Supplementary Information

Supplementary Information.

## Data Availability

The datasets generated during and/or analysed during the current study are available from the corresponding author.
