# Supplementary material for: Topography and relationship-specific social touching in individuals displaying body image disturbances
Source: Sci Rep. 2023 Aug 14;13:13198. doi: 10.1038/s41598-023-39484-w (PMC10425375; doi:10.1038/s41598-023-39484-w)
Supplement: Supplementary file 1 — Supplementary Information. [file 41598_2023_39484_MOESM1_ESM.docx]

**ONLINE SUPPLEMENTARY MATERIAL**

This document contains supplementary analyses, tables and figures accompanying the paper entitled:

**Topography and relationship-specific social touching in individuals displaying body image disturbances**

**Authors**: Ashleigh Bellard, Jyothisa Mathew, Wenhan Sun, Linda Denkow, Ali Najm, Despina Michael-Grigoriou, Paula Trotter, Francis McGlone, Merle Fairhurst, & Valentina Cazzato

**Demographics and self-report scales**

Table 1 displays the means and standard deviations for age and questionnaire subscales, which have been calculated separately for males and females. The third column in the table shows the results of a series of pairwise comparisons between the two gender groups (Bonferroni-corrected). Both groups did not significantly differ in age. However, both males and females differed regarding EDI-3 subscale scores, with males having marginally significantly higher drive for thinness and higher eating disorder composite risk. Males demonstrated significantly higher Perfectionism and Ascetism. Males and females did not differ in body dissatisfaction, Bulimia, low self-esteem, interpersonal alienation, emotional dysregulation, maturity fear, personal alienation, interpersonal insecurity, and interoceptive deficits. Females displayed higher levels of dysmorphic concerns as measured by the DCQ compared to males (see Table 1).

**Table 1.** Mean and standard deviation (in brackets) of demographics and self-report questionnaires scores for Males (*n= 22*) compared to Females (*n=47*).

|  | Males  (*n=22*) | Females  (*n= 47*) | Males vs. Females |
| --- | --- | --- | --- |
| **Age** | 29.55 (13.68) | 27.55 (10.04) | *t*(67) = .682, p =.498 |
| **EDI-3** |  | | |
| Drive for thinness | 13.00 (9.26) | 9.00 (7.35) | *t*(67) = 1.936, p = .057 |
| Body dissatisfaction | 20.59 (10.55) | 16.09 (11.07) | *t*(67) = 1.599, p = .115 |
| Bulimia | 15.59 (12.46) | 11.36 (9.94) | *t*(67) = 1.517, p = .134 |
| Low self esteem | 10.91 (7.85) | 9.26 (6.77) | *t*(67) = .899, p = .372 |
| Interpersonal alienation | 11.86 (7.97) | 9.34 (6.13) | *t*(67) = 1.445, p = .153 |
| Emotional dysregulation | 12.09 (11.31) | 9.98 (8.76) | *t*(67) = .849, p = .399 |
| Perfectionism | 12.82 (5.16) | 9.04 (4.53) | *t*(67) = 3.086, p = .003 |
| Ascetism | 13.59 (8.87) | 8.96 (7.72) | *t*(67) = 2.215, p = .030 |
| Maturity fear | 12.95 (7.45) | 11.94 (7.29) | *t*(67) = .537, p = .593 |
| Personal alienation | 13.64 (8.72) | 10.02 (7.33) | *t*(67) = 1.796, p = .077 |
| Interpersonal insecurity | 12.59 (6.49) | 10.68 (6.47) | *t*(67) = 1.142, p = .258 |
| Interoceptive deficit | 16.05 (11.59) | 11.47 (9.89) | *t*(67) = 1.695, p = .095 |
| EDRC | 49.18 (30.44) | 36.45 (22.86) | *t*(67) = 1.935, p = .057 |
| DCQ | 12.23 (5.07) | 15.66 (5.95) | *t*(67) = -2.337, p = .022 |
| ***Notes:*** *EDI-3* Eating Disorder Inventory 3; *EDRC* Eating Disorder Risk Composite*; DCQ* Dysmorphic Concern Questionnaire. | | | |

**Main Analyses**

**Imagined Social Touch ratings: Intimate vs. Social Body Regions**

The 4-way mixed ANOVA of Body Zone (Intimate vs. Social) × Relationship (Loved one vs. Acquaintance) × Group (High BIDS vs. Low BIDs) × Gender (Males vs. Females) on the soothing/unpleasantness ratings revealed a significant main effect of Body Zone [*F*(1,65) = 94.595, *p*< .001, *ηp^2^* = .593] and a main effect of Relationship [*F*(1,65) = 129.926, *p*< .001, *ηp^2^* = .667]. These main effects were further qualified by a significant 2-way interaction between Body Zone × Relationship [*F*(1,65) = 25.301, *p*< .001, *ηp^2^* = .280]. Post-hoc comparisons revealed that, when received by a loved one, touch to social regions was significantly rated as more soothing than touch to intimate regions (41.94 ± 4.43 vs. 29.47 ± 5.60, *p*< .001). On the other hand, when received by an acquaintance, touch to intimate regions was significantly rated as more unpleasant than touch to social regions (-47.47 ± 4.27 vs. -16.19 ± 4.06, *p*< .001). Furthermore, touch to intimate regions received from a loved one was rated as significantly more soothing than that received from an acquaintance, which was rated as unpleasant (29.47 ± 5.60 vs. -47.47 ± 4.27, *p*< .001). Touch to social regions were rated as significantly more soothing when provided from a loved one as opposed to an acquaintance which were rated as unpleasant (41.94 ± 4.43 vs. -16.19 ± 4.06, *p*< .001, see Fig. S1).


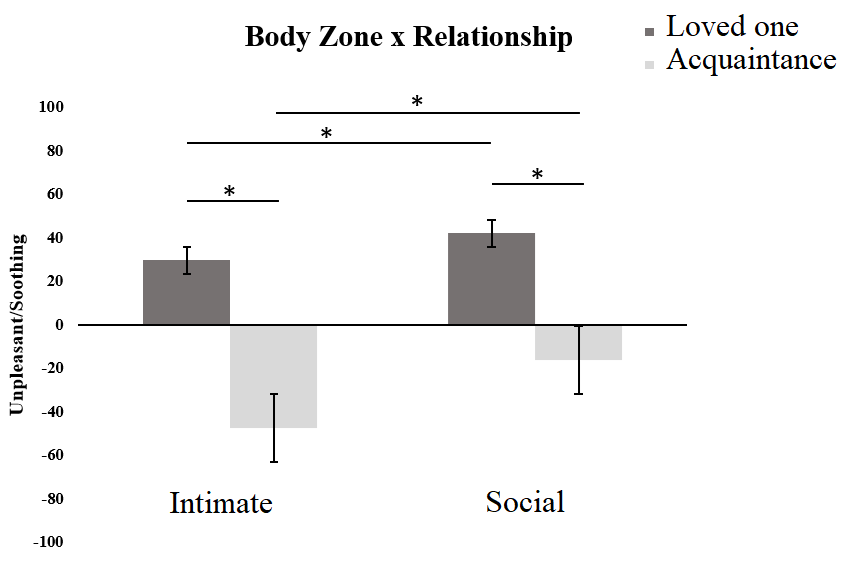


**Fig. S1:** Mean (M) and Standard Error of the Mean (S.E.M.) for soothing/unpleasant ratings for imagined touch delivered to each bodily regions (intimate vs. social) and for each relationship (loved one vs acquaintance).

A significant main effect of Gender was also revealed [*F*(1, 65) = 5.603, *p=*.021, *ηp^2^* = .079], which was further qualified by a significant 2-way interaction of Body Zone × Gender [*F*(1, 65) = 10.972, *p=*.002, *ηp^2^* = .144]. Post-hoc comparisons revealed that both females and males always rated touch to social areas as more soothing than touch received to intimate body areas. However, whilst no gender difference was found for touch received to social areas (females: 8.93 ± 3.67 vs. males: 16.82 ± 5.38, *p* = .21), on the contrary females rated touch to intimate areas as less pleasant than males did (females: -20.39 ± 4.05 vs. males: 2.39 ± 5.94, *p*< .001, see Fig. S2).


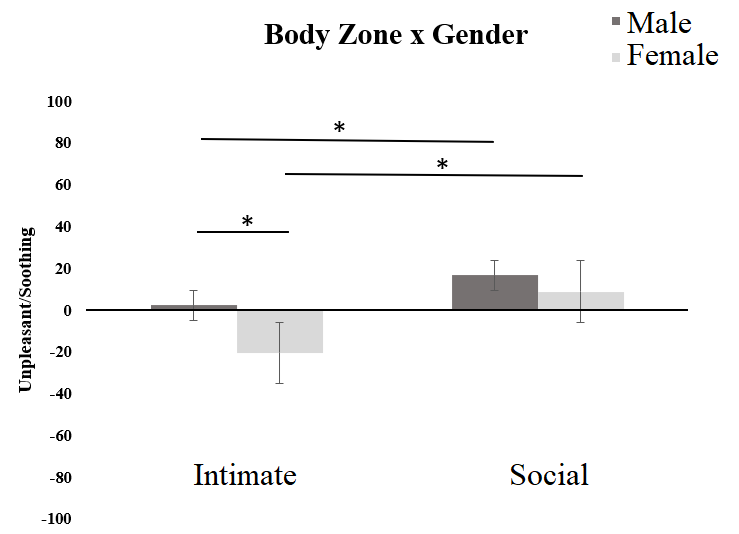


**Fig. S2:** Mean (M) and Standard Error of the Mean (S.E.M.) for soothing/unpleasant ratings for imagined touch delivered to each bodily regions (intimate vs. social) and for each gender group (male vs female).

Finally, no interaction between Group and Gender was significant, thus suggesting that females and males did not differ in their pleasantness ratings depending on their levels of BIDs. Finally, the remaining effects were all non-significant [All *Fs* < 3.656, *p* > 0.06, *ηp2* < 0.053].
